# Patient Empowerment: Apni Jung (Our Fight) against Rheumatoid Arthritis for South Asian Population

**DOI:** 10.31138/mjr.32.2.93

**Published:** 2021-06-30

**Authors:** Ailsa Bosworth, Shirish Dubey, Ade Adebajo, Arumugam Moorthy, Shivam Arora, Afshan Salim, Joti Reehal, Vibhu Paudyal, Monica Gupta, Kanta Kumar

**Affiliations:** 1 National Rheumatoid Arthritis Society, Maidenhead, United Kingdom; 2 Nuffield Orthopaedic Centre, Oxford University Hospitals NHS Foundation Trust, Headington, United Kingdom; 3 Faculty of Medicine, Dentistry and Health, University of Sheffield, United Kingdom; 4 Department of Rheumatology, University Hospitals of Leicester, Leicestershire, United Kingdom; 5 Bellevue Medical Centre, Birmingham, United Kingdom; 6 Institute of Clinical Sciences, College of Medical and Dental Sciences, University of Birmingham, Birmingham, United Kingdom; 7 Gartnavel General Hospital and Queen Elizabeth University Hospital, Glasgow, United Kingdom

**Keywords:** Education, ethnicity, patient support, chronic condition

## Abstract

Covid-19 has affected many populations in the UK, and ethnic minority communities in particular. People from ethnic minority communities living with long-term chronic diseases have shown to be less engaging with self-management and report having poor medication adherence. The main reason to this problem is the way information is delivered to non-English speaking patients. This editorial discusses an innovation to over this barriers in rheumatology practice.

Disparities in access to healthcare and ultimately in outcomes continue to be reported on ethnic grounds from around the globe.^1^ Although such inequalities have existed for many decades,^2^ the mortality and morbidity data from the Covid-19 pandemic has brought these issues to the fore.^3–6^ The increased predisposition to some rheumatological diseases^7^ and the continuing increased incidence of added comorbidity, such as coronary heart disease in South Asians, poses significant healthcare challenges,^8^ as does the need to begin addressing cultural differences in beliefs and behaviour at the very early stage.^9^ This is an area in which patient organisations such as the National Rheumatoid Arthritis Society (NRAS) can play a significant role and add great value to patient care.^10^ The continued observation of poor disease outcomes in South Asians suggests that in spite of the recognition of this need, there is still much to be done.^11–13^ If we are to achieve true equality of access to best rheumatological care for all, the rheumatology community needs to act by proactively addressing the barriers to care and treatment which many ethnic groups currently experience.^14^ COVID has shone a light on the inequity of access to healthcare generally faced by many ethnic communities and we need to grasp all opportunities to improve this imbalance.^4,6,15,16^

## APNI JUNG (OUR FIGHT) AGAINST RA

*Apni Jung* (our fight) against rheumatoid arthritis (RA) (www.nras.org.uk/apnijung) was launched by NRAS in collaboration with Dr. Kanta Kumar in 2016 at the BSR Annual Congress, based on the research over many years, demonstrating the barriers that people from ethnic communities face.^9,17–23^ They face challenges in accessing best care and treatment, and have culturally held health beliefs which may be different to the views of non-South Asians.^17^ It is widely recognised that provision of access to supported self-management, education, and appropriate information to help people manage their conditions is vital in a chronic, fluctuating, and evolving disease, such as RA.^10^ Accessible information and education can help people to cope day-to-day with the disease and improve their quality of life, through improving self-efficacy.^10^ Providing such information and educational resources in an accessible format which addresses issues around health literacy, facilitates self-management skills which can contribute to increased adherence to medication reduction in pain and fatigue, improved function, and also reduced utilisation of healthcare resources, is extremely important.^24,25^ Access to evidence-based information underpins these processes.^26^ Research has shown that people do not just need information about a disease, but also want to hear about real-life experiences of others with the same disease, as well as have access to peer support networks outside the healthcare setting.^25^ This influences how people make choices about treatments, how decisions are personalised, and, more importantly, how these are understood.^9^ The Internet is the obvious ‘go-to’ resource for anyone with a long-term condition, such as RA. It can provide information generically and quickly. However, without knowing which information is from reliable evidence-based sources, patients can easily be misinformed or find the information frightening. This is especially the case for minority ethnic populations, in whom information on rheumatology practice and care may not be presented in a culturally sensitive way, or necessarily in languages that are accessible to different ethnic populations.^12^ To address some of these challenges, we launched the NRAS *Apni Jung* web area on the NRAS website. Although people living in South Asian countries share genetic and cultural risk factors with South Asians living abroad, South Asians residing in the UK can differ in socioeconomic status, education, healthcare behaviours, attitudes, and illness perceptions which can affect their disease management and treatment outcomes.^2^ In rheumatology practice, departments in general produce written resources mainly in English.^24^ However, this is inaccessible to those who cannot read or write in English, or even in some instances, in their mother tongue.^12^ We know that literacy amongst a patient cohort in certain areas of the UK is a barrier to understanding written resource material given out in rheumatology clinics, even in those who speak English as a first language.^27^ Latif et al.^27^ found statistically significant improvement in knowledge of coronary artery disease, after a group of Bangladeshi women viewed a video on the topic. Videos would therefore be a better way by which to convey key health-related messaging in some patient populations. Up until 2016, there were limited resources for South Asian patients to access information in their own language and in a culturally meaningful way. The *Apni Jung* project aims to educate, empower, and engage ethnic minority populations. The initial success of the Apni Jung project has been highly encouraging, where clinicians have been signposting their patients. However, more needs to be done to implement this project in the heart of the South Asian communities and in routine clinical practice where there are patients of South Asian origin.

This effort requires a multidisciplinary approach, and a collective will to improve healthcare and outcomes in minority populations. The founders of *Apni Jung* have taken the next step of bringing together senior clinicians from a variety of specialties, and most importantly, patient advocates to form the “*Apni Jung* Advisory Board” to endorse and promote *Apni Jung*. We believe that strengthening the interface with primary care, pharmacy practice, and community and rheumatology healthcare professionals, will enable us to embed *Apni Jung* more successfully in the care pathways of rheumatology patients. The *Apni Jung* Board are aware that many people from ethnic communities do not always see patient organisations as having an important role to play in their care pathway. As a result, many do not seek information or support from patient organisations and rely solely on their doctor’s advice. To achieve a multidimensional approach to embed *Apni Jung* in the care pathway, the Advisory Board has pledged to raise awareness of this project much more widely across healthcare settings. This editorial highlights the important point that the way in which the rheumatology community currently provides information to patients of South Asian origin is not enabling and empowering them to self-manage their long-term conditions effectively. Sadly, poor knowledge and understanding of rheumatology diseases, appears to be very common amongst these populations, and *Apni Jung* is a freely available resource which health professionals should sign-post their patients to. In addition to conveying effective, evidence-based advice about disease recognition and management, we must include the vital information that *Apni Jung* can play a key role in empowering patients.

## CONCLUSION

We would like to encourage all rheumatology health professionals and primary and community care professionals involved in the care of South Asian populations to ensure that they sign-post patients to this important resource at point of diagnosis and indeed at any point in the patient’s journey. The rheumatology community serving ethnic minority population across the global can access this website for their patients: www.nras.org.uk/apnijung
